# *Alternaria* Toxins: Potential Virulence Factors and Genes Related to Pathogenesis

**DOI:** 10.3389/fmicb.2017.01451

**Published:** 2017-08-08

**Authors:** Mukesh Meena, Sanjay K. Gupta, Prashant Swapnil, Andleeb Zehra, Manish K. Dubey, Ram S. Upadhyay

**Affiliations:** Department of Botany, Institute of Science, Banaras Hindu University Varanasi, India

**Keywords:** *Alternaria* species, secondary metabolites, host-specific toxins, non-host specific toxins, conditionally dispensable chromosomes, pathogenicity

## Abstract

*Alternaria* is an important fungus to study due to their different life style from saprophytes to endophytes and a very successful fungal pathogen that causes diseases to a number of economically important crops. *Alternaria* species have been well-characterized for the production of different host-specific toxins (HSTs) and non-host specific toxins (nHSTs) which depend upon their physiological and morphological stages. The pathogenicity of *Alternaria* species depends on host susceptibility or resistance as well as quantitative production of HSTs and nHSTs. These toxins are chemically low molecular weight secondary metabolites (SMs). The effects of toxins are mainly on different parts of cells like mitochondria, chloroplast, plasma membrane, Golgi complex, nucleus, etc. *Alternaria* species produce several nHSTs such as brefeldin A, tenuazonic acid, tentoxin, and zinniol. HSTs that act in very low concentrations affect only certain plant varieties or genotype and play a role in determining the host range of specificity of plant pathogens. The commonly known HSTs are AAL-, AK-, AM-, AF-, ACR-, and ACT-toxins which are named by their host specificity and these toxins are classified into different family groups. The HSTs are differentiated on the basis of bio-statistical and other molecular analyses. All these toxins have different mode of action, biochemical reactions and signaling mechanisms to cause diseases. Different species of *Alternaria* produced toxins which reveal its biochemical and genetic effects on itself as well as on its host cells tissues. The genes responsible for the production of HSTs are found on the conditionally dispensable chromosomes (CDCs) which have been well characterized. Different bio-statistical methods like basic local alignment search tool (BLAST) data analysis used for the annotation of gene prediction, pathogenicity-related genes may provide surprising knowledge in present and future.

## Introduction

Fungal kingdom is very interesting in both useful and harmful point of view, which includes more than 1.5 million species, but only 100,000 species have been described, out of them 15,000 species cause disease in plants (Maharshi and Thaker, 2012). Due to increasing plants and fungal diversity, the complexity of pathogenic mechanism also increases between them on the morphologically level by forming a highly specialized structure of infections (Hawkswort, 1991; Horbach et al., 2011). Fungi produce various secondary metabolites (SMs) which affect their host plants at different stages of pathogenesis (Berestetskiy, 2008; Friesen et al., 2008a,b; Meena et al., 2015). The fungal pathogenic SMs are regarded as not essential for life, but their roles are quite versatile (Stergiopoulos et al., 2013; Pusztahelyi et al., 2015; Meena et al., 2016a). The genetically coded possibilities for the production of secondary metabolites, stimuli and the various phytotoxins generally predict the fungal-host plant interactions and pathogenic behavior of fungi.

The plant pathogenic fungi are divided into biotrophic, hemibiotrophic, and necrotrophic pathogens. These different pathogenic life styles require different molecular weaponry. Necrotrophic fungi infect and kill host tissue and extract nutrients from dead host cells. Biotrophic fungi colonize living host tissue and obtain nutrients from living tissue; whereas hemibiotrophic fungi display two phases during the infection process; first is an initial biotrophic phase followed by a necrotrophic stage (Lo Presti et al., 2015). Necrotrophic and hemibiotrophic fungal species basically show the contrasting mechanistic process of promoting disease, and many HSTs and proteins are the examples of effectors which fundamentally overlap (Condon et al., 2013). These life styles of plant pathogenic fungi provide general information about their interaction with the host, although the distinction between biotrophic and hemibiotrophic mode of action is still not so clear.

*Alternaria* species have shown different life styles i.e., from saprophytes to endophytes to pathogen (Thomma, 2003; Dang et al., 2015). They are very successful pathogenic genus that causes disease in large number of economically important plants, including apple, broccoli, cauliflower, potato, tomato, citrus, pear, strawberry, tobacco, etc. (Meena et al., 2016a). *Alternaria* creates large economic losses due to their host range and their worldwide distribution. Approximately, 300 species of genus *Alternaria* have been identified worldwide which includes *Alternaria alternata, Alternaria tenuissima, Alternaria arborescense, Alternaria brassicicola, Alternaria infectoria*, and *Alternaria solani* (Lee et al., 2015). These *Alternaria* species have been reported to cause diseases in nearly 400 plant species, in which *A. alternata* infects almost 100 plant species. It is also responsible for post-harvested diseases in various crops (Coates and Johnson, 1997; Woudenberg et al., 2015; Meena et al., 2017c; Sajad et al., 2017) causing asthma and infection of upper respiratory tract in humans (Kurup et al., 2000). The reasons behind pathogenicity are the production of diverse phytotoxins.

**Figure 1:**
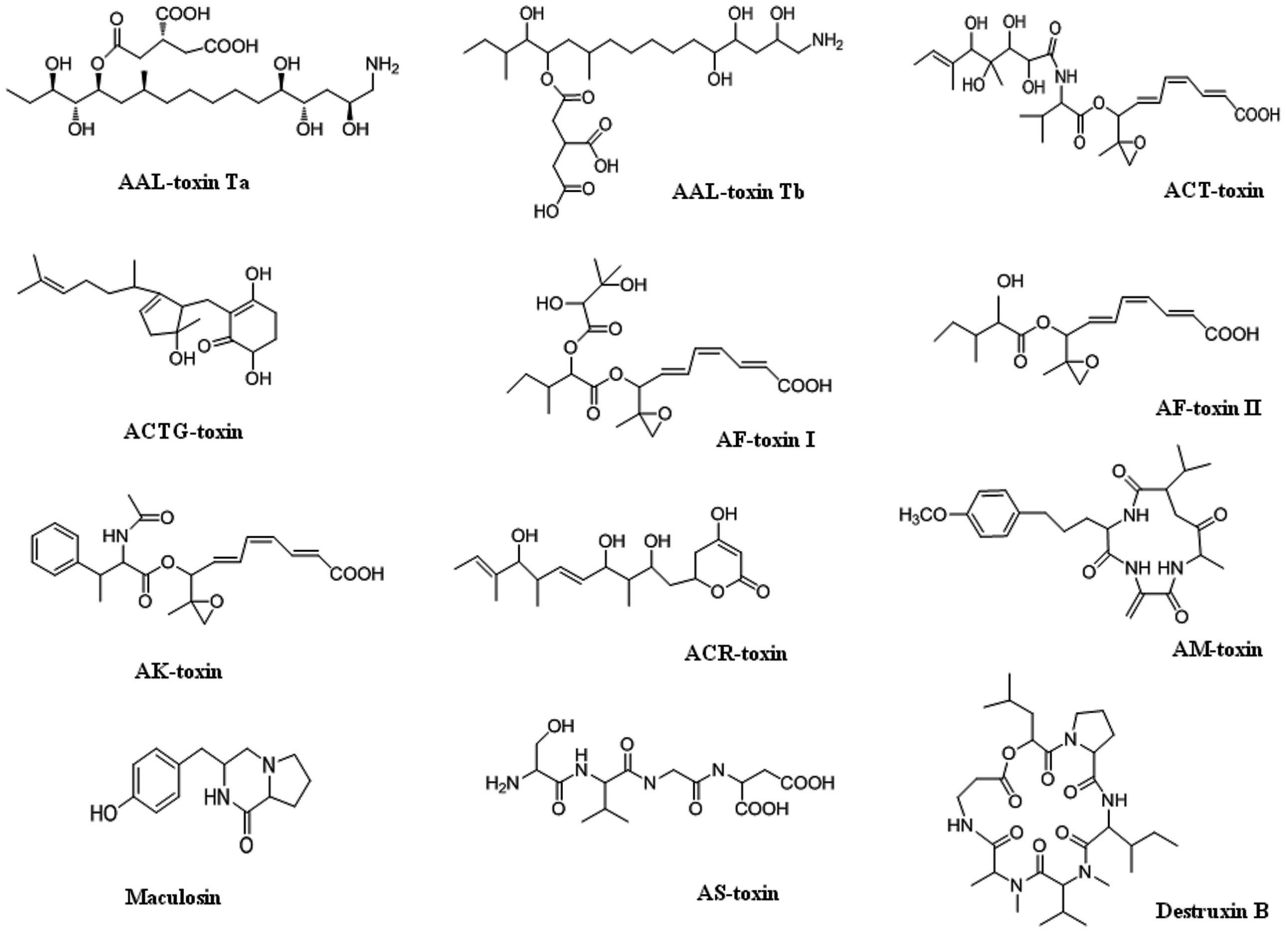
Chemical structures of HSTs produced by *Alternaria* species.

*Alternaria* mycotoxins have been frequently isolated and reported in fruits and vegetables, such as tomatoes, citrus fruits, Japanese pears, prune nectar, red currant, carrots, barley, oats, olives, mandarins, melons, peppers, apples, raspberries, cranberries, grape, sunflower seeds, oilseed rape meal, flax seed, linseed, pecans, melon, lentils, wheat, and other grains (Patriarca et al., 2007; Ostry, 2008; Logrieco et al., 2009; Andersen et al., 2015; Woudenberg et al., 2015; Meena et al., 2016a,b). More than 70 phytotoxins produced by species of *Alternaria* have been characterized, and include virulence factors that have both non-specific and specific host interactions. Several *Alternaria* SMs have been evaluated by the European Food Safety Authority (EFSA) as potentially causing risks to human health, including alternariol (AOH), alternariol monomethyl ether (AME), tenuazonic acid (TeA), altenuene (ALT), and tentoxin (TEN) [(EFSA Panel on Contaminants in the Food Chain (CONTAM), 2011; Rychlik, 2013)]. *Alternaria* produces host-specific toxins as well as non-host specific toxins (nHSTs). Generally, non-host-specific toxins have relatively mild phytotoxic effects, affect a broad spectrum of plant species and are thought to be an additional factor of disease alongside, for instance, penetration mechanisms and enzymatic processes. Although, they generally act as virulence factors and intensify disease symptom severity, they are not absolutely required for establishing disease since they are also toxic to plant species outside the host range of the pathogen. In *Alternaria*, many host-specific toxins have been identified, although the precise action of only a few has been studied in detail. Structures of different toxins are given in Figure 1. Brefeldin A (dehydro-), curvularin, tenuazonic acid, tentoxin, and zinniol are examples of non-host specific toxins that are produced by several *Alternaria* species (Thomma, 2003; Meena et al., 2017a,b).

## Pathogenicity of *Alternaria* species

The direct and indirect relationship hypothesis of pathogenicity proposed by Andrew et al. (2009), in which he expected that the genes of fungal genome database are responsible for pathogenicity and geographical distribution. The intensive study of disease pathogenesis via rDNA analysis has enabled phylogenetic classifications and investigations into the extent of DNA mutation (Lv et al., 2012). Further, there is no direct correlation observed between rDNA variant distribution and host-specific pathogenicity in toxins producing fungi (Koch et al., 1991). However, the pathogenic specialization of *A. alternata* might be controlled by some small number of genes which is helpful in HSTs toxin biosynthesis. Repeated DNA sequencing patterns of fungal supernumerary chromosomes suggest their different evolutionary history from essential chromosomes in the same genome and they may have been introduced into the fungal genome through horizontal gene transfer (HGT) from another species (Rosewich and Kistler, 2000). DNA-DNA reassociation and 16S rRNA sequence analysis are successful in bacterial taxonomy; therefore, these technologies are also applying in fungal taxonomy (Bruns et al., 2003). DNA-DNA analysis has suggested a close relationship between HSTs producing *Alternaria* fungal species and non-pathogenic *A. alternata* (Kuninaga and Yokozawa, 1987).

The question arises that how *Alternaria* does actually causes disease, and which symptoms result? The fungus on the attack of host plants secretes some hydrolytic enzyme during penetration process to gain entry into plant tissue and at that time plants also secrete some chemical compounds. In this way, there is a generation of cell fragmentation of plants and fungi. These oligosaccharides compounds can elicit broad host range defense responses that slow pathogen ingress. Thus, the rapid elicitation of plant's defense responses mandates the successful pathogenic fungi must have developed strategies to suppress the response or avoid the host's potential responses (Jackson and Taylor, 1996; Knogge, 1996). The effect of these fungi is mainly on genetics, mode of action, and biosynthesis. The low weight SMs have their target on the biochemical pathway, and their action that can have pleiotropic effects on host plant metabolism.

## Toxin production by *Alternaria* species

### Non-host specific toxins (nHSTs) by different *Alternaria* species

Approximately, 30 nHSTs secondary metabolites are known and characterized in which alternariol (AOH), alternuene (ALT), zinniol, tenuazonic acid (TeA), alteriol monomethyl ether (AME), brefeldin A (dehydro-), tentoxin (TEN), curvularin, and alterotoxin (ATX) I, II, III, are some of the known toxins produced by different *Alternaria* species (Andersen et al., 2015; Lee et al., 2015). Fujiwara et al. (1988) suggested that brefeldin A actions on Golgi-complex cause disassembly and act as an inhibitor of its secretion, while curvularin inhibits cell division by microtubule assembly disturbance (Robeson and Strobel, 1981) and tenuazonic acid inhibits process of protein synthesis (Meronuck et al., 1972). Zinniol disturbed membrane permeabilization (Thuleau et al., 1988), and tentoxin inhibits chloroplast photo-phosphorylation through binding to ATP synthase resulting in the ATP hydrolysis and ATP synthesis inhibition (Steele et al., 1978). Since, these toxins often target basic cellular processes, so these are regarded as potent mycotoxins. By UV/Vis and MS/MS spectra comparison, alternarionic acid, dehydrocurvularin, pyrenochaetic acid, and altechromone A were identified from species of *Alternaria* and other fungal SMs. Most of these are derivatives of AOH and TEN (Andersen et al., 2015).

### Host-specific toxins by different *Alternaria* species

On the basis of scientific observation, there are seven HSTs have been identified from *A. alternata*. These HSTs are families of closely related diverse group low molecular weight natural compounds (Tsuge et al., 2013). These families are: (1) Epoxy-decatrienoic acid (EDA)—AK-, AF-, and ACT- are the known toxic compound in this family, (2) Cyclic depsipeptide (cyclic tetrapeptide)—AM toxin also known as alternariolide, (3) Amino pento/polyketide (these are sphinganine analog compound)—AAL-toxin, and (4) Polyketide—ACR-toxin (syn. ACRL-toxin).

Interestingly, another HSTs is HC-toxin (a cyclic tetrapeptide), a well-known produced toxin by the plant pathogenic fungus *Cochliobolus carbonum*, has also been discovered in the species of *Alternaria jesenskae*, pathotype of *Fumana procumbens*, and the gene responsible for this toxin is *AjTox2* identified by genomic sequencing. Culture filtrate confirmed HC-toxin production through reverse phase HPLC and TLC assay (Wight et al., 2013), while its taxonomic identity confirmed by sequencing of ITS regions (Labuda et al., 2008).

These toxins are involved in the development of few destructive diseases. *Alternaria* pathotypes produce HSTs which are diverse ranged chemical compounds ranging from low molecular weighted peptides to cyclic peptides. Wolpert et al. (2002) reported that HSTs were biologically produced on their diverse specific plants species, so that it determines the host range of toxin-producing pathogens. Often, not all genotypes of a host plant species are sensitive to the toxin, and similarly, not all isolates of a pathogen species produce the toxin. Table 1 showing host-specific toxins (HSTs) produced by different species of *Alternaria*.

**Table 1:** Host-specific toxins produced by *Alternaria* species.

***Alternaria* species (Pathotype)**	**Disease**	**Host range (susceptible cultivar)**	**Gene**	**Host-specific toxins**	**Chemical characteristics**	**Target site of toxin**	**References**
*Alternaria alternata* f. sp. *lycopersici* (Tomato pathotype)	Alternaria stem canker of tomato	Tomato (Earlypack 7, First)	*ALT* genes	AAL-toxin Ta and Tb	Aminopentol esters	Aspartate carbamoyl transferase; sphinganine N-acltransferase	Bottini and Gilchrist, 1981; Abbas et al., 1994
*Alternaria alternata* f. sp. *citri tangerine* (Tangerine pathotype)	Brown spot of tangerine	Targerines and Mandarins (Dancy, Emperor, Minneola)	*ACTT* genes	ACT-toxin I and II	Epoxy-decatrienoic esters	Membrane protein	Nishimura and Kohmoto, 1983; Kohmoto et al., 1993
*Alternaria alternata* f. sp. *Fragariae* (Strawberry pathotype)	Black spot of strawberry	Strawberry (Morioka-16)	*AFT* genes	AF-toxin I, II and III	Epoxy-decatrienoic esters	Microsomal phospholipase A_2_	Nakatsuka et al., 1986; Lee et al., 2001
*Alternaria alternata* f. sp. *kikuchana* (Japanese pear pathotype)	Black spot of Japanese pear	Japanese pear (Nijisseiki)	*AKT* genes	AK-toxin I and II	Epoxy-decatrienoic esters	Sulfhydryl-containing molecules in membrane protein	Nishimura and Kohmoto, 1983; Nakashima et al., 1985; Izumi et al., 2012
*Alternaria alternata* f. sp. *citri jambhiri* (Rough lemon pathotype)	Leaf spot of rough lemon	Citurs rootstocks (Rough lemon)	*ACRT* genes	ACR(L)-toxin I	Terpenoid	Mitochondria	Gardner et al., 1985a; Akimitsu et al., 1989
*Alternaria alternata* f. sp. *mali* (Apple pathotype)	Alternaria blotch of apple	Apple (Red Gold, Starking)	*AMT* genes	AM-toxin I, II and III	Cyclic peptide	Membrane protein; chloroplasts	Kohmoto et al., 1987; Ueno, 1990
*Alternaria alternata* f. sp. *longiceps* (Tobacco pathotype)	Brown spot of tobacco	Tobacco	*ATT* genes	AT-toxin	–	Mitochondria	Kohmoto et al., 1987; Tsuge et al., 2013
*Alternaria alternata* (Spotted knapweed pathotype)	Black leaf blight of Knapweed	Spotted knapweed	–	Maculosin toxin	Cyclic peptide	Ribulose-1,5-bisphosphate carboxylase	Stierle et al., 1988, 1989
*Alternaria brassicae*	Gray leaf spot	*Brassica* species	*DtxS* genes	Destruxin A, B	–	Vacular H^+^-ATPase	Bains and Tewari, 1987; Pedras et al., 1999, Ma et al., 2010
*Alternaria alternata* (Sunflower pathotype)	Leaf spot of sunflower	Sunflower	–	AS-toxin I	Tetrapeptide		Liakopoulou-kyriakides et al., 1997
*Alternaria brassicicola* (*Brassica* pathotype)	Black leaf spot of *Brassica* species	*Brassica* species		AB-toxin	Protein		Chaube and Pundhir, 2005; Taj et al., 2015
*Alternaria brassicae*	–	–	–	ABR-toxin	–	–	Parada et al., 2008
*Alternaria panax*	–	–	–	AP-toxin	–	–	Quayyum et al., 2003

These HSTs produced several effects on a narrow species that serve as host to the fungi and are necessary for disease. In contrast to the HSTs, the dominant properties of less virulent small-spored species of *Alternaria* producing host-specific toxins are mainly due to strong selection pressure corresponding from modern monocrop agriculture and newly developed susceptible genotypes (Chou and Wu, 2002). Till now, 13 HSTs are identified by the different variants of *Alternaria* species and most of these variants are considered as *A. alternata* pathotypes (Table 1). In HSTs producing and nHSTs producing *Alternaria* species, all pathogenic species have some extra chromosomes, while these extra chromosomes are not carried by non-pathogenic species (Akamatsu et al., 1999; Akamatsu, 2004). The extra chromosomes are not required for normal growth but have an adaptive advantage in some habitats to the individual species. These are referred as conditionally dispensable chromosomes (CDCs).

### Mode of action and role of host-specific toxin

**Figure 2:**
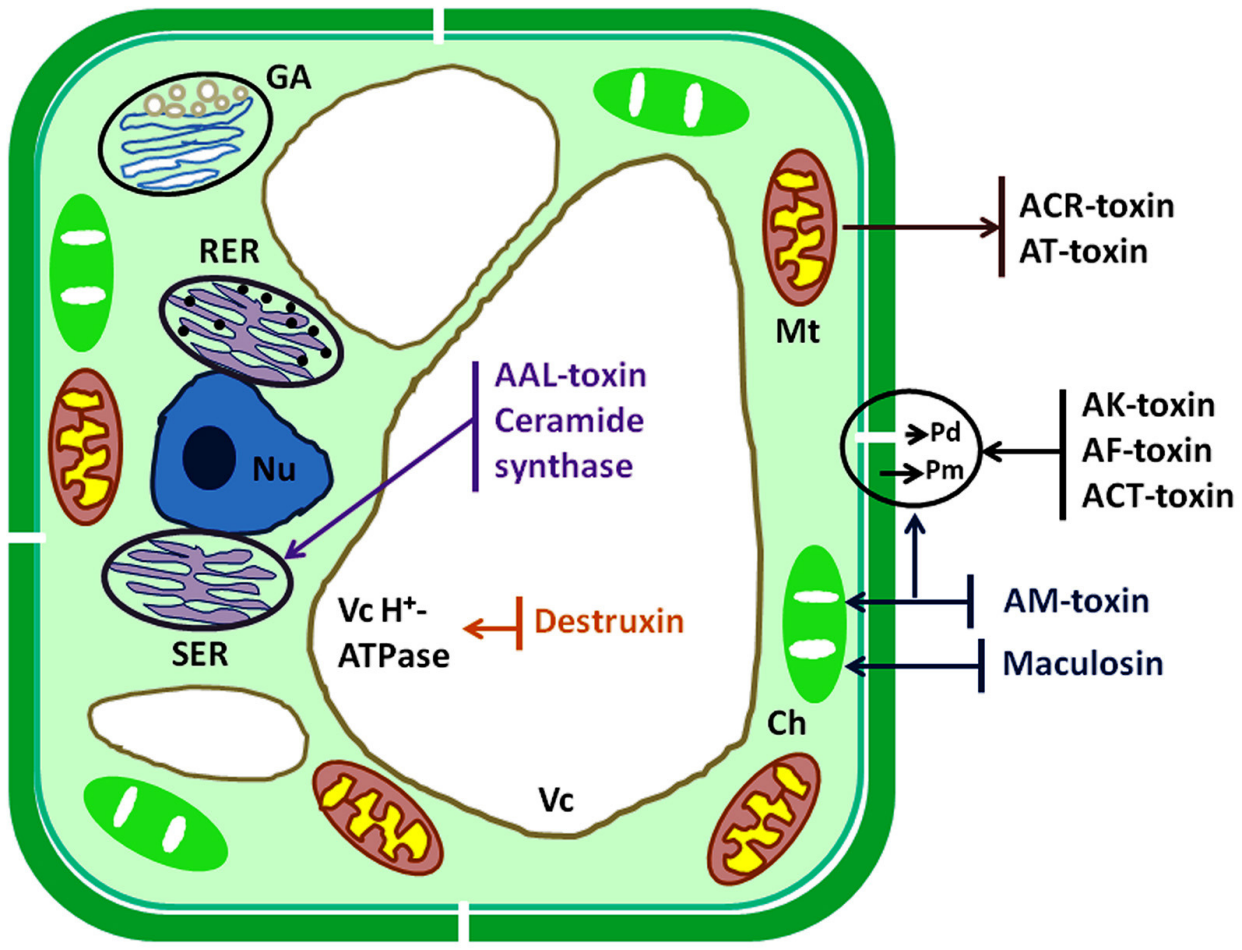
Schematic diagram of target sites of HSTs produced by *Alternaria* species. Ch, chloroplast; ER, endoplasmic reticulum; GA, Golgi apparatus; Mt, mitochondrion; Nu, nucleus; Pd, plasmodesma; Pm, plasma membrane; Vc, vacuole.

The plant pathogen fungi undergo several complex and crucial processing steps of pathogenesis viz. attachment to the plant surface, germination and formation of infectious structures, penetration, and colonization into host cell, which are crucially important steps to cause disease. Generally, plasma membrane, chloroplast, mitochondria, and some important enzymes are the inhibitory sites for the action of HSTs of *A. alternata* but the other target sites are ER, nucleus, vacuole, and Golgi bodies (Tsuge et al., 2013). All target sites are identified on the basis of different scientific studies (Tsuge et al., 2013; Meena et al., 2016a; Zhang et al., 2016). HSTs produce their effects on different targeted cell organelles and induce cell death in hosts (Figure 2). HSTs generally cause suppression of defense responses of genotype and also disturb other metabolic signaling pathway. HSTs have both properties such as necrosis and suppression of defense process of susceptible hosts (Wolpert et al., 2002). Kohmoto et al. (1989) observed the differences between pathogenicity and host cell death when interactions occur between AM-toxin producing isolates with apple and AK-toxin producing isolates with Japanese pear.

*Alternaria* HSTs play a vital role as an effector which determines the pathogenicity. Many scientists have reported that simultaneous treatment with HSTs and conidia of non-pathogenic *A. alternata* strains lead to the initiation of infection (Akimitsu et al., 1989; Yamagishi et al., 2006). The controversial finding of many scientists states that the gene silencing causing loss of HSTs production which leads to pathogenicity disappearance (Harimoto et al., 2008; Miyamoto et al., 2008). A hypothetical idea based on HST-receptor mediated model of pathogenesis was given by Pringle and Scheffer (1964). Before penetration fungal spp. produces a signal molecule to recognize host, and after that release fungal HSTs which specifically binds to host receptor site, and finally, these suppress the host defense against fungus and leads to cell death (Scheffer and Livingston, 1984; Kohmoto and Otani, 1991).

## Relation of HSTs, conditionally dispensable chromosomes (CDCs), and pathogenicity

The CDCs have some characteristic genes responsible for the production of specific HSTs. Several small chromosomes having HSTs genes and transposons like sequences in fungi which have been identified as CDCs, and are present in several small-spored *Alternaria* species (Hatta et al., 2002). Molecular studies suggested that these HSTs biosynthetic gene clusters reside on a single small chromosome which is <2 Mb in size (Akamatsu et al., 1999; Tsuge et al., 2013). These HSTs genes are located on CDCs as gene clusters and control the production of the toxins viz. AM-toxin from apple, AF- toxin from strawberry, AK-toxin from Japanese pear, ACT-toxin from tangerine, and AAL-toxin from tomato pathotypes and so on (Hu et al., 2012).

## Predictions of CDCs genes

Two methods are commonly used to predict the residential CDCs genes function, (I) use of BLAST against national center for biotechnology information (NCBI) non-redundant database, Pfam (Bateman et al., 2004), and NCBI for functional domains search (Marchler-Bauer et al., 2005), and (II) scanning to identify transcription factors, pathogenicity related genes, *PKS* genes, *NRPS* genes, and P450 transporters (Hu et al., 2012). Hu et al. (2012) used marker assisted contigs identification in *Alternaria* to identify CDCs genes which carrying two *Alt1* and *AaMSAS* cluster of toxin biosynthetic genes (Yamagishi et al., 2006). Among 209 predicted proteins sequencing data, some CDCs genes were identified and characterized, in which 31 having PKS domains and 2 proteins have high modulator protein domains: KS-AT-KR-ACP on CDC_141 and KS-AT-DH-ER-KR-ACP on CDC_165 and remaining 29 PKS protein carrying over two ACPs (Acyl carrier proteins) domains, 7 NRPS domains proteins were predicted having 3 Enterobactin domains, 2 Bacitracin domain, 1 Pyochelin domain and 1 CDA1 domain, 7 protein for P450 monooxygenase, 24 for transcription factor and 37 were identified for pathogenicity during plant-pathogen interactions (Baldwin et al., 2006; Proctor et al., 2009; Hu et al., 2012).

Most recently, another method was applied to genome annotation of *Alternaria* and comparison data has facilitated functional genomics studies of the fungus in the context of plant and human pathogenicity (Dang et al., 2015). Dang et al. (2015) also provided sequential genome's annotation and comparisonal data of 25 *Alternaria* species which were analyzed by using multiple computational and comparisonal genomes incorporated tools. Researchers analyzed the sequences through multiple annotation modules including repetitive sequence annotation, gene prediction, protein function and domain structure, while whole genome alignment and homology analysis methods were performed for comparative genomics (Koonin and Galperin, 2003; Bihon et al., 2016). They used InterPro database (Hunter et al., 2012) and Pfam (Finn et al., 2013) for protein domain and family annotation, Blast2GO (Gotz et al., 2008) and InterPro for gene annotation, signal P (Bendtsen et al., 2004), WOLF-Psort (Horton et al., 2007) and Phobius (Kall et al., 2007) for signal peptides, TMHMM (Krogh et al., 2001) for trans-membrane proteins, BLAST search against PHI-base (Winnenburg et al., 2008) for pathogenicity-related candidate genes, CAZY database (Cantarel et al., 2009) and dbCAN (Yin et al., 2012) for active carbohydrates enzymes, BLAST based homology searches and Allerdictor for the identity of allergens (Dang and Lawrence, 2014), batched BLAST search tools from MEROPS database (Rawlings et al., 2012) for protease annotation and SMURF were used for SMs (Khaldi et al., 2010). They reported the *Alternaria* annotation and comparisonal genomes data sequences to Ensembl database schema using a self-developed tool (EnsImport). EnsImport supports multiple standard file formats such as FASTA, AGP, GFF3, and XMFA, and outputs from widely-used tools such as BLAST, InterPro, RepeatMasker, OrthoMCL, and Blast2GO.

## Genetics of *Alternaria alternata* produced HSTs

HSTs and nHSTs produced by *Alternaria* species play an important role as virulence factors during plant pathogenesis. Many known and unknown genes are responsible for the production of these SMs (Dang et al., 2015). The gene sequences identified as CDCs are known as extended families of transposon like sequences (Hatta et al., 2006). The term commonly used is HGT, which is the movement of genetic material without any recombination (Syvanen, 1985). The fungal HGT, the movement of plasmids, mycoviruses, gene clusters, transposable elements and sometime whole chromosomes have expressed between the individual species (Rosewich and Kistler, 2000). HGT in *Alternaria* occurs frequently because of its wide host range transportable pathogenicity of chromosome may increase pathogens adaptation to the environment, asexual reproduction, and loss of CDC in the absence of a host and cause reduction of carrying extra genome content (Hu et al., 2012). The genetic analysis observation indicated that the fungal toxin protein virulence patterns expected to match with the host sensitive proteins in some manner which is identified in some fungal races (Friesen et al., 2007, 2008b). So, directly or indirectly both the pathogen HST gene and a host susceptible gene interaction between their genes products are required for disease, which inverse situation of gene-for-gene interaction (Wolpert et al., 2002). HSTs genes functions as virulence factors with largely additive effects on disease development and used to influence host plant physiology (Horbach et al., 2011).

HSTs producing *A. alternata* fungus becomes a good model organism for fungal developmental studies due to its characterized effects on the host plant. Each pathotype toxin and disease on a particular host can be identified by its specificity and can be distinguished by their necrotic symptoms that developed after inoculation with the disease causing pathotype or after HSTs treatment by pathotype (Izumi et al., 2012). Different studies have suggested that RNA silencing and homologous recombination-mediated gene destruction are essential for particular HSTs productions and pathogenicity (Tanaka and Tsuge, 2000; Izumi et al., 2012). Molecular assessment basis of HSTs have identified the gene clusters for pathogenic specialization (Tsuge et al., 2013).

## Important HSTs produced by *Alternaria* species

### AAL-toxin

AAL-toxins are chemically propane 1,2,3-tricarboxylic acid (PTCA) which is esterified to 1-amino-11,15-dimethylheptadeca-2,4,5,13,14-pentol. These are structurally sphingosine and sphinganine analogs (Bottini and Gilchrist, 1981; Brandwagt et al., 2001). The scientist identified five types of AAL-toxin related molecules viz. sphingosine (TA), phytosphingosine (TB), sphinganine (TC), tetra-acetyl-phytosphingosine N- Lignoceroyl-d (TD), and L- sphinganine, each consisting with two isomers (Caldas et al., 1994). TA and TB analogs of AAL-toxins showing related specific action and showing 30–400 times higher activity than the other form analogs TC, TD, and TE (Caldas et al., 1994). So, these two toxins are taken into consideration and referred as AAL-toxins. These toxins are produced by *Alternaria alternata* f. sp. *lycopersici*, a pathogen causing stem canker in tomato (*Lycopersicon esculentum*) which exhibits high degree of host specificity and plays a major role in pathogenesis by causing leaf necrosis (Prasad and Upadhyay, 2010). TA type of toxin is most active and highly produced which has molecular mass of 522 kb. AAL-toxin sensitivity and insensitivity to plant tissue have been revealed (Spassieva et al., 2002). The necrotic symptoms of AAL-toxin can be detected from a susceptible line of detached leaflets (Brandwagt et al., 2001). Gilchrist (1997) assumed that AAL-toxin inhibits the activity of aspartate carbamoyl transferase (ACTase) on disruption of pyrimidine metabolism but it could not be confirmed experimentally. AAL-toxins isolated from *Alternaria alternata* f. sp. *lycopersici* inhibited ceramide synthase and induced programmed cell death (PCD; Michaelson et al., 2016).

Kawaguchi et al. (1991) observed that ethanolamine (EA), phosphoethanolamine (PEA) and five other related chemicals accumulation occur in AAL-toxin treated leaves. It was also found that cell free ACTase preparation of homozygous susceptible and homozygous resistant genotypes of host and non-host specific sources express differential AAL-toxin sensitivity. Generally, AAL-toxins have its effect on mitochondria but their exact target site is still uncertain. The possible AAL-toxin biosynthetic pathway was in under investigation because EA and PEA are primary and secondary intermediate metabolites of biosynthetic pathways.

Orolaza et al. (1992) suggested that ^14^C labeling of ethanolamine to susceptible AAL-toxin treated leaf discs have shown strong inhibition of EA incorporated into phosphatidyl ethanolamine (PtdEA). Therefore, phospholipid pathway involved enzymes were suggested as potential biochemical targets for AAL-toxins. AAL-toxins have structural similarities to fumonisins because AAL-toxins have one PTCA and fumonisins with two PTCA side chains esterified to aminopentol back bones (Brandwagt et al., 2000). Gilchrist (1998) referred both AAL-toxin and fumonisin collectively as sphinganine analog mycotoxins (SAMS) due to their structural and functional similarities, and toxicity to plant and mammalian cells. They also show inhibitory action to sphingolipid biosynthesis and induced PCD in both plant and mammalian cells (Abbas et al., 1994; Spassieva et al., 2002, 2006; Tsuge et al., 2013). The sensitivity and disease resistance in tomato to AAL-toxin are regulated by *Asc1* (Alternaria stem canker resistance gene 1) gene locus which encodes a host recognition factor (Egusa et al., 2009). These plant-pathogen interactions come under direct or indirect interaction between AAL-toxin and *Asc* locus products in which *Asc* action is linked to sucrose transport, ethylene biosynthesis, pyrimidine metabolism, and cell death (Moussatos et al., 1994).

According to Zhang et al. (2011), AAL-toxin induced PCD in tomato leaves is promoted by both jasmonic acid and ethylene by disrupting sphingolipid metabolism. While, the experimental finding of Akamatsu et al. (1997) states that AAL-toxin deficient REMI mutants are non-pathogenic to sensitive tomato plants. AAL-toxins also induce apoptotic-like responses. The induced PCD was taken place by DNA laddering, TUNEL-positive cells and the formation of apoptotic like bodies (Tsuge et al., 2013). AAL-toxin induced PCD involves ceramide signaling and cell cycle disruption (Wolpert et al., 2002). The physiological effects of AAL-toxins represent development of necrotic lesions on fruits and leaves, inhibition of *in-vitro* development of calli, pollen, roots and shoots, and also reduce the viability of protoplasts and suspension cells (Ismaiel and Papenbrock, 2015).

### AM-toxin

AM-toxin is other type of HSTs, which is responsible for causing Alternaria blotch on apple, a worldwide distributed disease. It is cyclic depsipeptide of alternariolide (Ueno et al., 1975, 1977). This type of cyclic depsipeptide chemical structural compound is also found in other toxins of plant pathogens i.e., HC-toxin from *Cochliobolus carbonum* race1 (Gross et al., 1982) and tentoxin from *Alternaria tenuis* (Mayer et al., 2001). Cyclic peptides are synthesized by non-ribosomal pathways by large multifunctional enzymes called non-ribosomal peptide synthetase (NRPS), and also by polymerase chain reaction (PCR) based cloning with primers having highly conserved domains of fungal *NRPS* genes (Keller et al., 2005; Tsuge et al., 2013). *AMT1* and *AMT2* are two biosynthetic genes for AM-toxin (Johnson et al., 2000; Harimoto et al., 2007). *AMT1* encodes non-ribosomal peptide synthetase attaching with four catalytic domains, which is responsible for activation of each residue in AM-toxin whereas *AMT2* encodes an aldo-keto reductase enzyme required for biosynthesis of 2-hydroxy-isovaleric acid i.e., one of the AM-toxin residues (Harimoto et al., 2007, 2008). Chloroplasts are the important cellular organelles which serve as the primary site for AM-toxins. On the basis of AM-toxin acting site and known pathogenesis, it is identified that *Alternaria alternata* f. sp. *mali* pathogen may secrete AM-toxins that act on the susceptible leaf cells causing tissues damage (Zhang et al., 2015).

*Alternaria alternata* f. sp. *mali* (apple pathotype) has small chromosomes having <1.9 Mb size which is not found in non-pathogenic strains of *A. alternata*. It suggests strongly that *AMT1* resides on small chromosome of 1.1–1.8 Mb of apple pathotype strains (Johnson et al., 2001). The apple pathotype mutant strains have 1.1 Mb CDCs encoding *AMT* genes which are responsible for AM-toxin biosynthesis and its pathogenicity to susceptible apple strains (Johnson et al., 2001). According to Covert (1998), fungal supernumerary chromosomes are not required for growth but it has advantages for colonizing certain ecological niches (Harimoto et al., 2007).

EST (expressed sequence tag) analysis of *AMT* genes encoded on 1.4 Mb chromosome in the apple pathotype strain IFO 8984 was performed to identify the structure, function, and origin of *Alternaria alternata*, in which CDCs responsible for HSTs biosynthesis (Harimoto et al., 2007). Harimoto et al. (2007) generated a cDNA library from AM-toxin producing culture and identified 80 unigenes from 40,980 clones with 1.4 Mb chromosome probe from 196 ESTs. The sequence analysis of these genes showed that most of the small chromosomes encoding these genes with unknown function and also most of the genes expressed at remarkably low level under testing condition. Comparison of the transcription levels of the genes in toxin-producing and non-producing cultures identified 21 genes, including *AMT1* and *AMT2*, that were up-regulated (>10 fold) in toxin producing cultures. Sequence analysis suggested that the up-regulated genes include candidates for novel AM-toxin biosynthetic genes. Disruption of three genes, *AMT2, AMT3*, and *AMT4* also upregulated in toxin-producing cultures IFO8984 having multiple copies of the genes in the genome, and all showed similarities in structures (Tsuge et al., 2013).

### AF-toxin

AF-toxins are other types of *A. alternata* produced HSTs toxins having <2 Mb sized CDCs encoding *AFT* genes (Hatta et al., 2002). AF-toxin produced by *A. alternata* has three related molecular species types viz. AF-toxin I, II, and III. AF-toxin I is highly toxic to both strawberry and pear (Nishimura and Nakatsuka, 1989; Tsuge et al., 2013). Another, AF-toxin II is toxic to only pear while toxin III is highly toxic to strawberry and slightly to pear (Maekawa et al., 1984). AF-toxin I and III are valine derivatives of 2,3-dyhydroxy-isovaleric acid and 2-hydroxy isovaleric acid respectively, while AF II is an isoleucine derivative of 2-hydroxy valeric acid (Tsuge et al., 2013).

Hatta et al. (2006) concluded structure of AF-toxin, on the basis of 1.0 Mb chromosomal strain of NAF8 and found 2–7 copies of 20 AFT regions. They also found many transposon-like sequences and most of which were inactive transposon fossils. On cellular level, AF-toxin affects plasma membrane of susceptible cells and causes a sudden increase in loss of K^+^ after a few minutes of toxin treatment (Park and Ikeda, 2008). These toxin-induced dysfunctions of plasma membrane which were confirmed by microscopic study with no other cell organelles (Tsuge et al., 2013). Electrophysiological studies of AF-toxin showed that plasma membrane becomes irreversibly depolarised (Namiki et al., 1986; Otani et al., 1989). A polarization occurs mostly in the respiration-dependent component of membrane potential which is sustained by H^+^ pump. AF-toxin possibly affects the plasma membrane H^+^ATPase (Tsuge et al., 2013). However, no direct effect of AF-toxin was observed on isolated susceptible host cell plasma membrane ATPase activity (Akimitsu et al., 2013).

### AK-toxin

AK-toxins are the esters of 9,10-epoxy 8-hydroxy 9-methyldecatrienoic acid (EDA) produced by Japanese pear pathotype of *A. alternata* and was first reported in the Japanese pear black spot disease (Nakashima et al., 1985; Nakatsuka et al., 1986; Tsuge et al., 2013). EDA is an intermediate for toxin biosynthetic pathways. H^3^-labeled EDA when added in a growing liquid culture of the Japanese pear pathotype strain, converted to AK-toxin. Tanaka et al. (1999) used restriction mediated-integration transformation (REMI) to isolate AK-toxin-minus (or AK-toxin lack) mutant (Akimitsu et al., 2013). They observed that mutants affected essential AK-toxin biosynthetic genes and structural and functional analysis of clones containing tagged site represented six AK-toxin biosynthetic genes i.e., *AKT1, AKT2, AKT3, AKT4, AKTR*, and *AKTS1* (Tanaka and Tsuge, 2000). *AKTR* encodes transcription regulator having a zinc binuclear cluster DNA binding domain, a protein type of fungal Zn(II)2 Cys6 family (Tanaka et al., 1999; Tanaka and Tsuge, 2000) and other fungal genes encoding proteins showed similarity with many enzymes (Tsuge et al., 2013). So, it acts as regulatory genes for AK-toxin biosynthetic enzymes.

Japanese pear pathotype strains also have multiple copies of functional or non-functional homologous of the *AKT* genes (Tanaka et al., 1999; Tanaka and Tsuge, 2000). *AKTS1* also found unique in this pathotype in DNA blot analysis when these techniques used to assess AKT homologous distribution in *A. alternata* pathogen (Tanaka et al., 1999). These *AKTS1* genes were also present in the tangerine and strawberry pathotype and all three pathotype share common genes required for EDA biosynthesis.

REMI techniques used for AKT protein and *AKT1* gene tagging to mutagenize which is required for biosynthesis of AK-toxin and pathogenicity of the Japanese pear pathotype (Tanaka et al., 1999; Wolpert et al., 2002). Another experimental observation of Masunaka et al. (2000) stated that *AKT1* and *AKT2* were also present in tangerine and strawberry pathotype. The two other *AKT3* and *AKTR* genes are also required for AK-toxin biosynthesis. *AKT1, AKT2, AKT3*, and *AKTR* and all their homologous are present on a single chromosome (Tanaka and Tsuge, 2000). Imazaki et al. (2010) reported that enzymes of AK-toxin biosynthesis are localized in peroxisomes and peroxisomal targeting signal type1 (PS1)- like tripeptides at their C-terminal ends. Mutation in *AaPEX6*, which encodes a peroxin protein required for peroxisome biogenesis from the Japanese pear pathotype. Lack of this peroxisome function, AK-toxin production and pathogenicity become loss completely.

### ACR-toxin (Syn. ACRL-toxin)

ACR-toxin, a polyketide of long fatty acid (Gardner et al., 1985a,b) causes rough lemon leaf spot disease. The target site of this toxin is mitochondria which makes it dysfunctional (Kohmoto et al., 1984; Akimitsu et al., 1989). ACR-toxin causes uncoupling of oxidative phosphorylation and causes leakage of NAD^+^ cofactor from tricarboxylic acid cycle (TCA) and loss of membrane potential (Akimitsu et al., 1989).

The clustered biosynthetic genes of ACR-toxin have generally occurred on <2 Mb small sized chromosomes (Ito et al., 2004; Miyamoto et al., 2008, 2009, 2010; Ajiro et al., 2010; Izumi et al., 2012). Masunaka et al. (2005) observed that lemon pathotype strains also carried a small chromosome of ~1.5 Mb which is correlated with the toxin production and pathogenicity to rough lemon.

**Figure 3:**
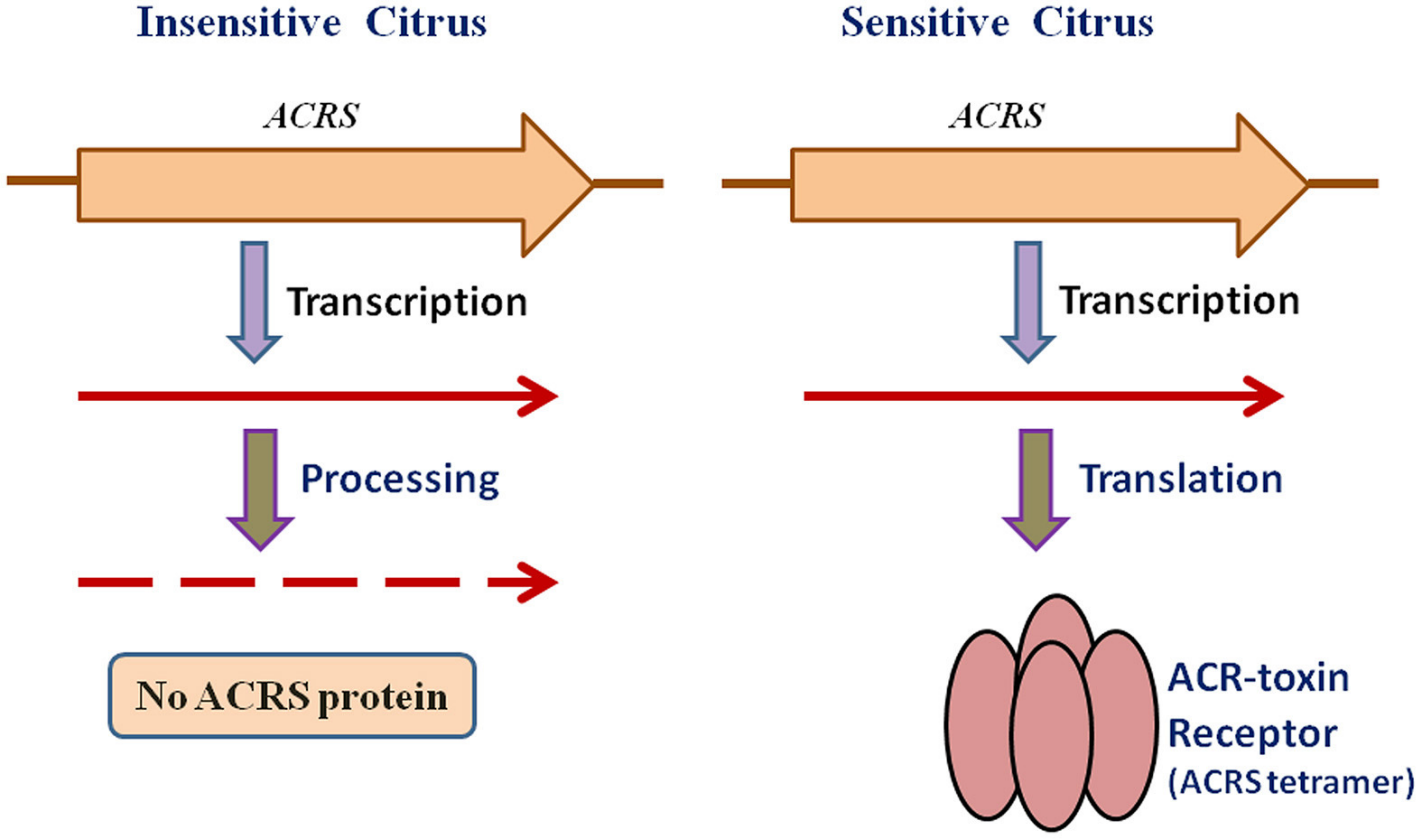
Mechanism of ACR-toxin sensitivity controlled by receptor transcript processing in mitochondria.

To investigate the relationship of ACRS to sensitivity to ACR-toxin and hence susceptibility to *A. alternata* rough lemon pathotype, Ohtani et al. (2002) sequenced this region of the mitochondrial genome from resistant cultivars and 13 species of citrus and found that the regions in the resistant citrus are identical to that of rough lemon (Ohtani et al., 2002). However, examination of ACRS transcripts demonstrated that sensitivity to the toxin is not controlled by the presence or absence of ACRS but rather by post-transcriptional modification of the ACRS transcripts (Figure 3). The peptide encoded by ACRS was detected by immunoblotting only in rough lemon mitochondria, but not in toxin-insensitive citrus mitochondria, and the peptide appeared to consist of sodium dodecyl sulfate (SDS)-resistant oligomers that have been reported for many pore-forming transmembrane proteins (Figure 3; Ohtani et al., 2002).

*ACRTS1* and *ACRTS2* are the two genes required for ACR-toxin production. Hutchinson and Fujii (1995) classified *ACRTS2* as types I enzyme, is a single large and multifunctional polypeptide having all necessary enzymatic domains for multiple cycles of condensation and β-keto processing. *ACRTS1* has more than three copies in the rough lemon pathotype genome. However, combination of both homologous recombination-mediated gene disruption and RNA silencing functionally suppressed all paralogs. Therefore, this gene is required for ACR-toxin biosynthesis and pathogenicity (Izumi et al., 2012), but there are some limitations of homologous recombination-mediated gene disruption. It is difficult to disrupt entire duplicated and multiple paralog functional copies of genes. RNA silencing solves this problems by knockdown all transcripts from all functional copies in HST- producing *A. alternata* (Miyamoto et al., 2008).

RNA silencing method was first applied to tangerine pathotype HST biosynthesis gene *ACTT2*, a polyketide synthase (PKS) which is similar to rough lemon pathotypes. This is silenced by transforming the pathotype strain with a plasmid construct expressing hairpin ACRT2 RNA. The ACRT2-silenced transformants in which ACRT2 transcripts were not detectable, ACR-toxins were lost the production and pathogenicity, indicating that this gene encodes a PKS essential for ACR-toxin biosynthesis and hence pathogenicity (Izumi et al., 2012).

### ACT-toxin

Polyketides are the largest families of SMs synthesized by fungi, microbes and plants (Anand et al., 2010). ACT-toxin is one of the types of polyketides HSTs of tangerine pathotype. *A. alternata* causes brown spot disease (Timmer et al., 2000; Miyamoto et al., 2009). ACT-toxin action appears to be complex in the cell and the primary site of action for this toxin is plasma membrane (Kohmoto et al., 1993; Miyamoto et al., 2008). Kohmoto et al. (1993) detected ACT-toxins ACTT1 and ACTT2 from purified germinating conidia fluids of tangerine pathotype and expected that it participates in the biosynthesis of ACT-, AK- and AF-toxins due to their related common chemical structure (Tanaka et al., 1999; Masunaka et al., 2000, 2005).

Molecular analysis by Masunaka et al. (2000, 2005) suggests that both ACT-toxins are present on a single chromosome with a size of 1.1–1.9 and ACTT2 toxins exists in the genome as multiple copies form. Harimoto et al. (2008) used repeated round of homologous recombination-mediated gene disruption which is essential to knockout all copies of a particular target gene but it is difficult to disrupt entire copies of *ACTT1* genes. Homologous gene disruption has some limitations like lack of selectable marker for multiple transformants, low efficiency etc. Harimoto et al. (2008) also overcome these limitations by RNA silencing and beneficial to knock down all functional *ACTT1* genes. Since, RNA silencing induced double stranded RNA (dsRNA) post-transcriptional gene silencing phenomenon. It is cleaved by Dicer (a nuclease of the RNA III family) into small interfering RNAs (siRNA) to a ribonucleo-protein complex [RNA-induced-silencing-complex (RISC)] (Bernstein et al., 2001; Hammond et al., 2001). RISC recognizes and degrades homologous mRNAs by complementary base pairing (Elbashir et al., 2001). The RNA silencing used vectors generate hairpin structure of RNA. This structure have sense and antisense sequenced target gene with an intron sequence-based hairpin head spacer which are effective and relatively stable for sequencing, which will be useful for HSTs genes function in the other pathotype of *A. alternata* as well as other filamentous fungi producing HSTs (Liu et al., 2002; Kadotani et al., 2003; Miyamoto et al., 2008).

RNA silencing has never been demonstrated in *Alternaria* spp., so Isshiki et al. (2003) had constructed a vector for generating hairpin RNA of the green fluorescent protein (GFP) gene in *A. alternata*. The silencing genes were over-expressed with green fluorescence protein for determining the gene function. In addition, they analyzed the function of *ACTT2* gene in ACT biosynthesis. The *ACTT2* gene having multiple copies and high sequence identity were silenced by introducing a vector generated *ACTT2* hairpin RNA. The sequence identity of *ACTT2* and *ACTT1* is 98.8% while *ACTT2* homologs identity from dual toxin producing *A. alternata* strain also expressed similarity of 99% for *ACTT2* and 98.9% *ACTT3* (Miyamoto et al., 2008).

## Conclusions

Genomic and transcriptomic comparisons are now taken in use to obtain genetic features of fungal pathogen to survive successfully in various stressful ecological habitats and to survive itself in different pathogenic life styles. The deep knowledge of the plant pathogen may be helpful for the production of new resistant variety of plant cultivars against different biotic stresses. Environmental factors such as heat or drought also affect the plant-fungal interactions as well as SMs production.

Toxins are recognized as important determinant of pathogenicity in different species of *Alternaria*. Host-specific toxins of *Alternaria* spp. plant pathogens play an important role in pathogenesis and could be applied as selective agents in *in-vitro* selection at the cellular level for disease resistance. The role of a toxin as a disease determinant is proved by the occurrence of the toxin in infected plants and the ability of the toxin alone to elicit at least part of the symptoms of the disease.

Genome sequencing and genome analysis comparison of pathogenic variation in the different species of *Alternaria* have fascinated to understanding of its evolutionary relationship with other fungi and identification of pathogenicity associated candidate genes, and provide important information for understanding its virulence variation and mechanism under its interaction with the host.

During last decades, the molecular events at an ever-increasing rate applying to know the gene data history and cellular processes responsible for the specific role of toxins. In future, these combined analytical approaches with new insight and revised concepts of new research in fungal genetics and biochemistry will provide a surprising knowledge of SMs corresponding biosynthetic genes and its effect on cellular processes.

## Author contributions

MM provided the general concept and drafted part of the manuscript. MM, SG, PS, and AZ wrote the manuscript. MD and RU also helped in preparation of the manuscript as required by the journal guideline. All authors revised and approved the manuscript.

### Conflict of interest statement

The authors declare that the research was conducted in the absence of any commercial or financial relationships that could be construed as a potential conflict of interest. The reviewer AMR and handling Editor declared their shared affiliation, and the handling Editor states that the process nevertheless met the standards of a fair and objective review.

## Abbreviations

SMs: Secondary metabolites
HSTs: Host-specific toxins
nHSTs: Non-host specific toxins
CDCs: Conditionally dispensable chromosomes
HGT: Horizontal gene transfer
PCD: Programmed cell death
PCR: Polymerase chain reaction
TCA: Tricarboxylic acid cycle
BLAST: Basic local alignment search tool
NCBI: National center for biotechnology information.
